# Gender-inclined Young Age Glycosuria: Contribution to Late Age Chronic Renal Diseases, Type 2 Diabetes Mellitus and Cardiovascular Diseases

**DOI:** 10.24248/eahrj.v7i1.713

**Published:** 2023-07-12

**Authors:** Mohamed O Mng'agi, Ambele M Mwandigha, Erasto V Mbugi

**Affiliations:** a Muhimbili University of Health and Allied Sciences, Dar es Salaam, Tanzania.

## Abstract

**Background:**

Chronic kidney diseases (CKD), Type 2 Diabetes Mellitus (T2DM) and cardiovascular diseases (CVDs) are the recent worldwide late age chronic conditions that could be a consequence of renal glycosuria during childhood. This study aimed at determining the extent of glycosuria in secondary school students to obtain information that could be predictive of the situation in late age life of Tanzanians living in Mkuranga District.

**Methodology:**

This was school-based cross-sectional study that was conducted in assenting and consenting 800 students from July to October 2019 in Mkuranga district, Pwani-Tanzania. Socio-demographic information was collected using well-structured questionnaires while weight and height were measured using beam balance and tape measure, respectively. Dipstick strip was used to determine urine glucose on clean catch mid-stream urine collected specimens.

**Results:**

From a total of 800 enrolled students, 0.6% (5/800) had glycosuria from whom 80% were males and 20% (1/5) were females (*p = 0.37*). The proportion of glycosuric males was 4 folds higher than that found in females. While height, body mass index (BMI) and waist–hip circumference ratio were associated with renal glycosuria (*p < 0.05*), other factors showed no association (*p > 0.05*).

**Conclusion:**

Despite the low proportion (0.6%) of glycosuria in this study, the contribution of young age renal glycosuria to old age CKD, T2DM and CVDs cannot be ruled out with males being more prone than females. Thus, it signals for consideration of regular screening for glycosuria in the school health programmes as an intervention strategy to prevent potential late age chronic disease complications.

## BACKGROUND

Renal glycosuria is among conditions, usually asymptomatic, with consequences at a later age in life. The consequences may be huge and serious diseases that may include, chronic kidney disease (CKD) and type 2 diabetes mellitus (T2DM).^1,2^ Renal glycosuria is not gender-biased, although males seem to be more prone to the condition than females.^3^ It is suggested that the renal threshold falls with age in some individuals and rare in children; also infants have immature organs (kidney) such that it functions at low efficiency.^3^ Renal glycosuria is a benign condition in which affected individuals may not have any complaints, but seldom may experience episodes of hypovolemia and hypoglycaemia.^4^ In patients with renal glycosuria, blood sugar (glucose) is abnormally excreted in the urine due to inappropriate functioning of the renal tubules.^4^ Renal tubules are primary components of the filtering units of the kidneys (nephrons). Glycosuria affected patients excrete glucose in urine while blood glucose concentrations remain normal or relatively low.^5^

Renal glycosuria affects individuals in various parts of the world, regardless of the age, sex or race. Glycosuria has been studied in non-white racial groups, South African, Indians, Malays, and Bantu. It has been found that glycosuria of any type is rare in children with non-diabetic glycosuria being more common in men than in women.^6,7^ The overall prevalence of glycosuria among Indians and Africans is 8.6% and 8.0%, respectively. Factors such as kidney disorders, high intake of carbohydrate feeding, body mass index, waist-hip circumference ratio, genetic, family history of diabetes, lack of body physical exercise, and age are the risk factors for renal glycosuria.^4,5,7^ In Tanzania there are no currently reported studies providing information on the prevalence of renal glycosuria particularly in non-diabetic children, but there are few studies that relate diabetes and glycosuria.^8^ With no doubt, data on the prevalence of glycosuria and its associated factors in non-diabetic secondary school students is limited. This study therefore, aimed to determine the prevalence of renal glycosuria and its associated factors among secondary school age students with the view of obtaining information that could be predictive of the CKD, T2DM and CVCs in late age life of Tanzanians.

## MATERIALS AND METHODS

### Study Area

This was a school-based cross-sectional study conducted from July to October 2019 at Mkuranga district in Pwani, Tanzania. Mkuranga district is one of the six districts of Pwani region in Tanzania located between 7° 16' 12” south and 39° 12' east with an elevation of 88 metres (289 feet). It is bordered to the north by Temeke district of Dar es Salaam, to the east by the Indian Ocean, to the south by Rufiji District, and to the west by Kisarawe District. Mkuranga districts is administratively divided into 18 wards with a population of approximately more than 187,428. The district possesses 41 secondary schools.

### Sample Size, Study Population and Sampling Procedure

The sample size was calculated using the Kish Leslie formula, n = z^2^p(1-*p*)/e^2^, where ‘n’ stands for sample size, ‘z’ stands for the level of confidence (1.96) at 95% confidence interval, ‘*p*' is the proportion of glycosuria estimated at 8.0% ^7^ and ‘'the margin of error ‘e’ was taken at 1.88%. Based on these calculations, the minimum required sample size was thus estimated to be 800 subjects, the number that was believed to provide sufficient and reliable information.

After knowing the sample size, in July to October 2019, a school-based cross-sectional study of 800 students from 10 out of 41 randomly selected secondary schools (included are: Dundani, Kiimbwanindi, Kiparang'anda, Kisiju Pwani, Lukanga, Mkamba, Mwarusembe, Mwinyi, Vianzi and Vikindu secondary schools) at Mkuranga district in Pwani, Tanzania was carried out. A total of 80 students which were obtained by dividing a total sample size (800) to the number of secondary schools (10) and subjects purposively included in the study. Thus 80 (800/10) subjects were recruited from each secondary school, each class level (I-IV) generating 20 students for enrolment. Through simple random sampling 10 males and 10 female students were then selected from a group of males and females students per class level for inclusion.

### Data Collection

To collect socio-demographic information that included age, sex, weight and height, waist and hip circumferences, carbohydrate feeding habit, physical body exercise and genetic family history of diabetes mellitus, well-structured questionnaires were used. A measuring tape was used to measure height, waist and hip circumferences while a calibrated beam balance was used to measure weight from 609 assented students whose parents/guardians consented for the participation in the study and 191 consented students (older or equal to 18 years old). Both measuring tape and beam balance were tested for accuracy and validity before using for taking measurement from the students. Dipstick strip was used to determine urine glucose on clean catch mid-stream urine collected specimens.

### Quality Control

All strips and urine containers were stored at room temperature and dry place as per manufacturer's recommendation. To ensure the validity of the test strips, a strip was dipped into 100g/dl glucose solution and sterile water, then the readings were justified as expected.

### Statistical Analysis

STATA version 15.1 software was used to enter data, clean and for statistical analysis. Continuous variables (such as age, weight, height, body mass index, waist-hip circumference ratio and waist height ratio) were summarized as mean and standard deviations. Categorical variables such as sex, carbohydrate feeding habit, physical body exercise and genetic family history of diabetes mellitus were described as proportions. Univariate analysis was performed to determine factors associated with glycosuria by using Fisher's exact test for categorical variables and student's t-test for continuous variables. A p-value <0.05 was considered significant.

### Ethics Approval and Consent to Participate

Ethical clearance with Ref. No. DA.287/298/01A/ was obtained from MUHAS Research Ethics Sub-Committee of the Senate's Research and Publications Committee of the Muhimbili University of Health and Allied Sciences (MUHAS). Permission to conduct the study was obtained from Director of Mkuranga District Council after explaining the purpose of the study and having common understanding. Confidentiality of the study participants was ensured using codes instead of participant's names. Participants were given feedback on the urinary test results and those who were found to have abnormal glucose presentation in the urine (glycosuria) were advised accordingly, to prevent progress of the condition and the measure for rectification to normal urine glucose.

## RESULTS

### General Characteristics of Study Population

A total of 800 school students with a mean age (years), body mass index (kg/m2), weight (kg), height (m), waist-hip circumference ratio and waist-height ratio of 16.3±1.6, 21.4±5.0, 49.1±7.9, 1.5±0.1, 0.76±0.03, and 46.3±4.8, respectively (Table 1) were enrolled in the study. In this study equal proportion of males 400 (50%) and females 400 (50%) were recruited. High proportion of the study participants were using carbohydrate (77.1%) and most of them were practicing physical body exercise (52.7%). Out of 800 study participants, 2 (0.3%) had family history of diabetes mellitus (Table 2).

**TABLE 1:** Mean Distribution of Continuous Independent Factors Among Secondary School Students (N = 800)

Variable	Mean ±SD
Age (Years)	16.3±1.6
Weight (Kg)	49.1±7.9
Height (m)	1.5±0.1
Body Mass Index	21.4±5.0
Waist-hip circumference ratio	0.76±0.03
Waist Height ratio	0.46±0.48

**TABLE 2:** Distribution of Categorical Independent Factors Among Secondary School Students (N = 800)

Variable	Total participant	Proportion (%)
Sex		
Female	400	50
Male	400	50
Quantity of carbohydrate feeding		
Low	183	22.9
High	617	77.1
Physical body exercise		
Low	422	52.7
High	378	47.3
Genetic family history of Diabetes Mellitus		
No	798	99.7
Yes	2	0.3

### Glycosuria among Secondary School Students

**FIGURE 1:**
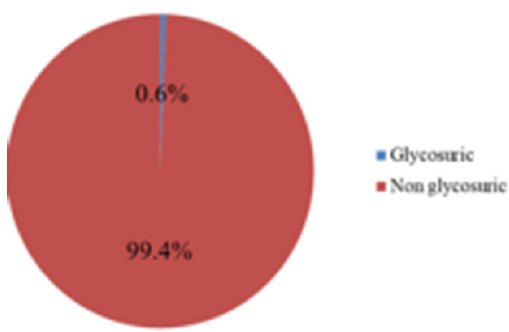
Proportion of Positive Reactor Students for Glycosuria

Screening for glycosuria among secondary school students revealed an overall prevalence of 0.6% (5/800) (Figure 1). Out of 5 glycosuric students 4 (80%) were males and 1 (20%) was a female (Table 4). Therefore, the proportion of glycosuric males was 4 folds higher than that found in females.

Smaller Number of Students Reacted to Glycosuria Test

### Predictors of Glycosuria among Secondary School Students

In this population, the mean height (*p* = 0.002), BMI (*p* < 0.001) and waist–hip circumference (*p* < 0.001) seemed to be the factors significantly associated with glycosuria (Table 3). The urinalysis results showed that 250 mg/dl of glucose were contained in urine sample of three subjects amongst four males with glycosuria with one male having as much as double the concentration (500 mg/dl) compared to others. The only female with glycosuria had urine glucose concentration of 250 mg/dl.

**TABLE 3:** Predisposing Categorical Independent Factors Associated with Glycosuria

Variable	Total (Mean±SD) N = 800	Glycosuric (Mean±SD n=5	Non glycosuric (Mean±SD) n=795	P-value
Age (Years)	16.3±1.6	16.2±1.5	16.3±1.6	0.87
Weight (Kg)	49.1±7.9	53.2±13.2	49.0±7.9	0.24
Height (m)	1.5±0.1	1.3±0.2	1.5±0.1	0.002
BMI	21.4±5.0	29.8±4.1	21.4±5.0	<0.001
Waist-hip ratio	0.76±0.03	0.90±0.04	0.76±0.03	<0.001
Waist Height ratio	0.46±0.48	0.50±0.47	0.46±0.48	0.07

P-value of less than 0.05 that indicates statistically significant association (Fisher's exact test)

**TABLE 4:** Predisposing Continuous Independent Factors Associated with Glycosuria

Variable	Total N (%)	Glycosuric n (%)	Non glycosuric n (%)	P-value
Sex				
Female	400 (50.0)	1 (0.2)	399 (50.2)	0.37
Male	400 (50.0)	4 (0.8)	396 (49.8)	
Quantity of carbohydrate feeding				
Low	183 (22.9)	0 (0.0)	183 (23.0)	0.59
High	617 (77.1)	5 (100.0)	612 (77.0)	
Physical body exercise				
Low	422 (52.7)	3 (0.6)	419 (52.7)	1.00
High	378 (47.3)	2 (0.4)	376 (47.3)	
Genetic family history of Diabetes Mellitus				
No	798 (99.7)	5 (100.0)	793 (99.7)	1.00
Yes	2 (0.3)	0 (0.0)	2 (0.3)	
Overall	800 (100)	5 (0.6)	795 (99.4)	

P-value of less than 0.05 that indicates statistically significant association (Fisher's exact test)

## DISCUSSION

This study explored the prevalence of renal glycosuria in potentially prone population of Secondary School students from 10 schools, in Mkuranga district. In principle, the results revealed presence, although in small proportion, of glycosuria in the study population with 0.6% of subjects having the condition. The proportion of subjects with glycosuria in the studied population, despite being small, provides an alert of prevailing situation in the area. The prevalence of glycosuria obtained in this study does not differ much from what was reported in a stuy conducted in Nigeria^3^ which revealed a prevalence of 0.7%. However, in their study, Bassey and his colleague enrolled a large group of adolescents (1008 students) with different standard of living of whom some were in private and others in public schools. But in our study, the subjects had relatively similar standard of living and life style. The result does not seem to differ much as both studies recruited subjects with similar age ranges. In another study conducted on glycosuria and diabetes mellitus, A study conducted in South India^12^ reported lower prevalence of glycosuria of 0.038% among 10,513 Indians children aged between 3 and 20 years. As compared to the current prevalence of 0.6%, a higher prevalence of 8.0% was reported in Cape Town South Africa.^7^ The difference could be due to the difference in lifestyle between the South African and Tanzanian populations. The difference in prevalence could also be due to the larger sample size studied with age groups including children below 5 years who are less likely to develop diabetes unlike in the present study that involved only adolescents. Difference in geographical location of the study participants might be a cause of the difference in the prevalence of glycosuria noted in the Indian and Tanzanian studies. A prevalence of 0.8% was reported in 2004 in China among women aged 40-70 years.^13^ The slight difference between the prevalence might be attributed to the difference in participants enrolled, because the previous study involved only females who are older with potential for developing pre-diabetic or T2DM than the subjects in the current study whose focus was the group of school children aged below 18 years old. The larger sample size in the previous study might have contributed to the increase in chances of finding the subjects with a condition compared to the current study. In addition, the difference in nutritional intake in Mkuranga indigenous (which is mostly sugar containing food like cassava, potatoes, bananas and maize) and other parts of the world may lead to variation of prevalence between this study and others.

This study found the mean BMI of 29.8±4.1 in subjects with glycosuria the value which is relatively higher than those without glycosuria 21.4±5.0. This finding is similar to studies conducted elsewhere^14, 15^ which found a higher prevalence of hyperglycaemia, glycosuria and diabetes among those with higher BMI. It is known that BMI is directly related to obesity which is a major risk factor for glycosuria and T2DM, because obesity influences insulin resistance leading to hyperinsulinaemia and finally to T2DM.^16^ The mean Waist Hip Ratio (WHCR, 0.90±0.04) of all the subjects with glycosuria was higher than WHCR (0.76±0.03) of those without glycosuria. These findings compare with those of the study conducted elsewhere.^17^ The variable WHCR resembles the BMI which is also directly related to obesity, which is a major risk factor for T2DM and glycosuria.^18^ The findings are also supported by a study done elswhere.^19^ Our study revealed significant association between glycosuria and BMI (*p =* .001). However, might happen that BMI and WHCR to be insignificant risk factors for glycosuria, that may result due to delayed development of insulin resistance (in the presence of adequate beta cell function) which can manifest later in adult life if appropriate measures to control obesity are not instituted. Since may result into significant association between glycosuria and obesity, this indicates that although BMI and obesity is a risk factors for glycosuria and DM, not all obese children may develop glycosuria and DM.^20^ Height is associated with glycosuria (*p =* 0.002) in that, it can affect the body mass index and the obesity. Shorter individuals are more prone to high BMI that may lead to glycosuria. A study conducted in Sub Saharan Africa^19^ suggested that obesity and high BMI which is affected by height of an individual is much contributing to DM and glycosuria.

## CONCLUSION AND RECOMMENDATIONS

The study demonstrated prevalence of renal glycosuria of 0.6% among secondary school students with male (80%) being more prone to the condition than female (20%).

We observed the factors such as height, BMI and WHCR as the risk factors for renal glycosuria among secondary school students. The findings showed gender-influence as regards to glycosuria, inclining more to males than females. These findings provide an insight for future regular screening and checkup for glycosuria in children at lower age and adolescence to serve as an early potential preventive measure for metabolic syndromes. The findings obtained could influence the reported findings as they are important determinants for external validity to this study. Further studies particularly including more diverse clustering of participants from young age to elders of 50 years of age are needed to further explore the factor-condition association. This can provide reliable information for future strategic prevention and control of potential etiology, consequently limiting future occurrence of glycosuria and other chronic complications. The study however, has provided clues on the prevalence of the condition with gender-bias, something that could be closely watched in future interventions.

### Limitation of the study

One of the limitations of this scholarly study is that it was a cross-sectional study that aimed to screen for glycosuria as a potential contributor to late age chronic renal diseases, type 2 diabetes mellitus and cardiovascular diseases. In this context the study did not dig up on other factors that could contribute to the condition including measurement of blood glucose levels (both glycated and random indicative of poor blood glucose control) and renal function tests to check for potential nephropathies that might contribute to renal damages. Nevertheless, the dipstick strip measurement for urine glucose on clean catch midstream urine collected specimens was deemed sufficient to provide at least a picture on glucose levels per individual.
